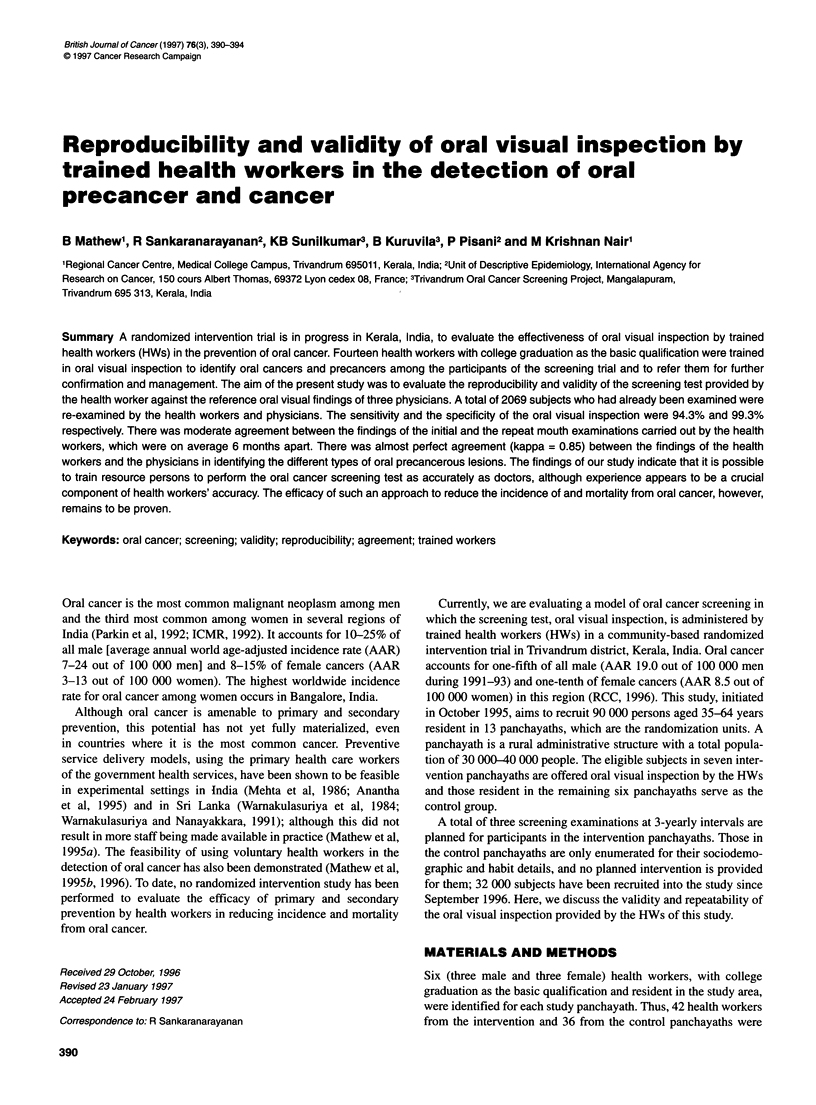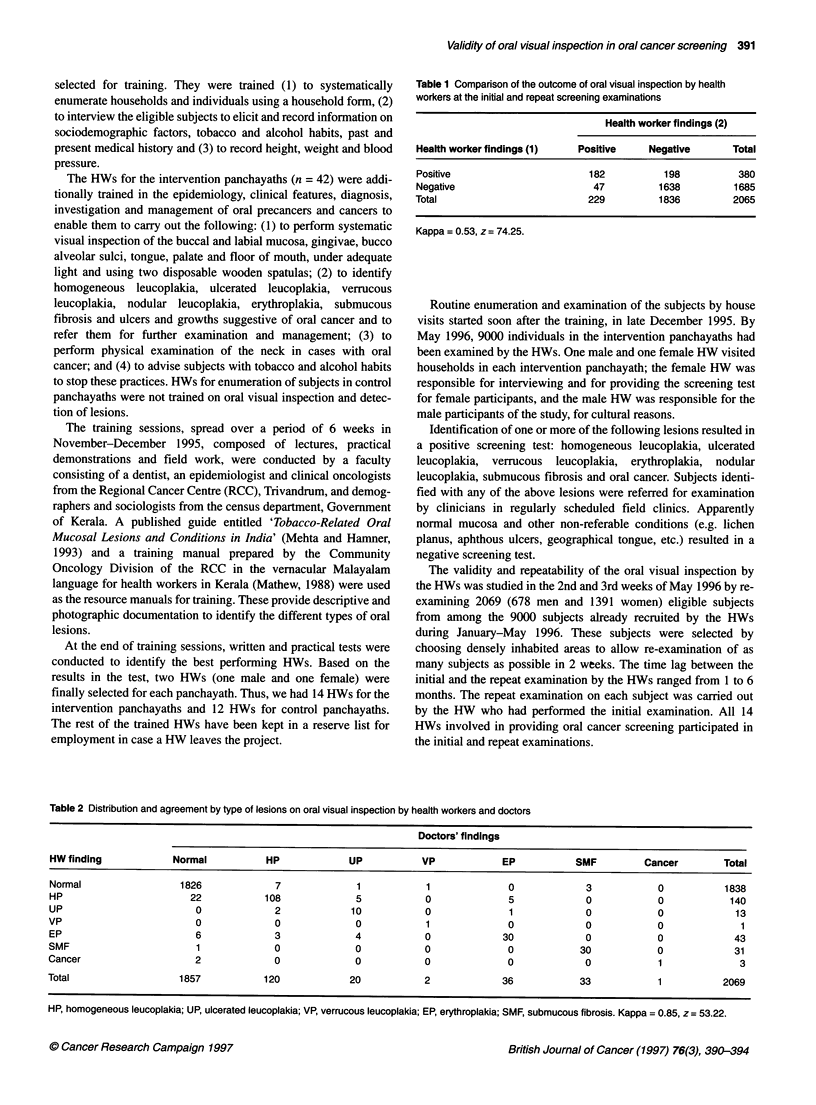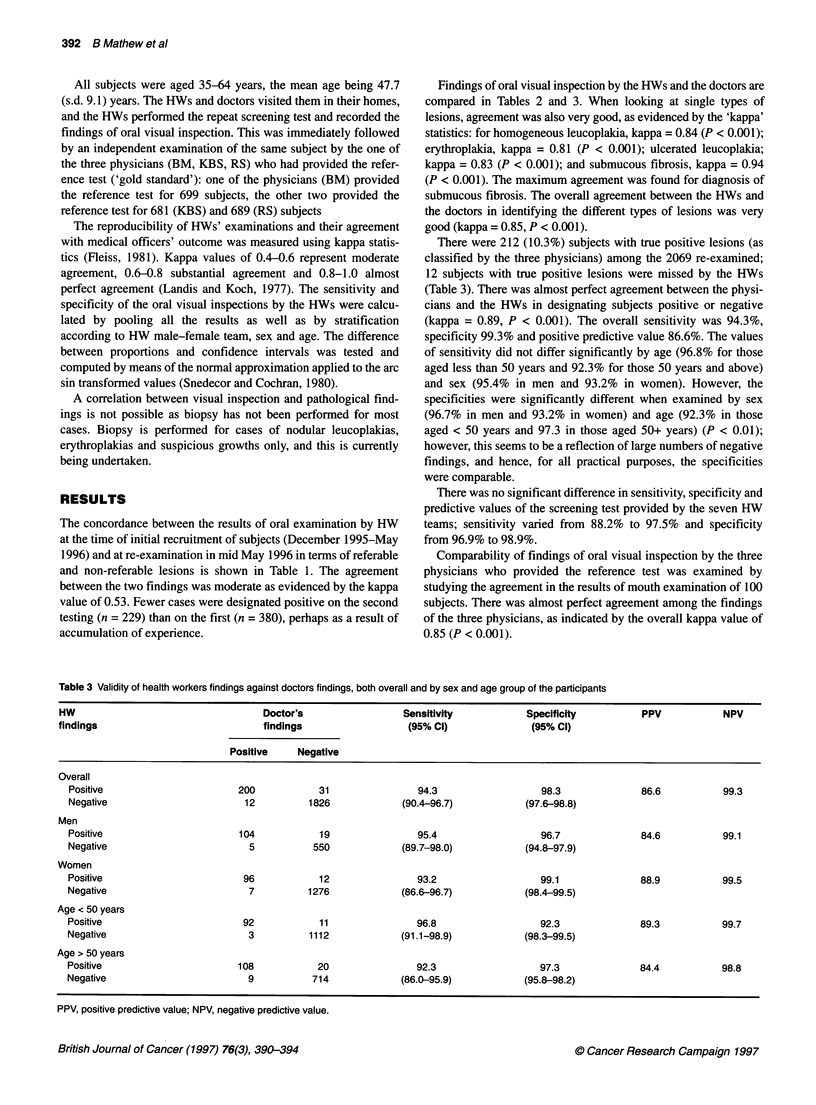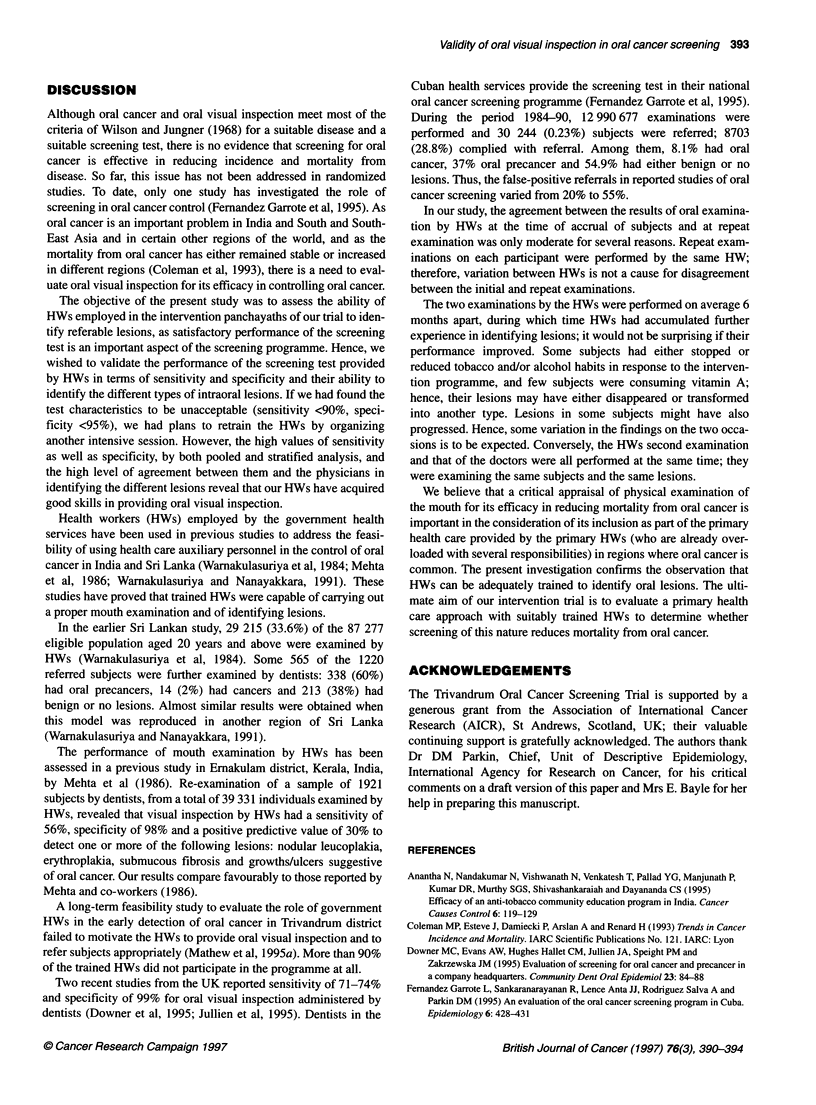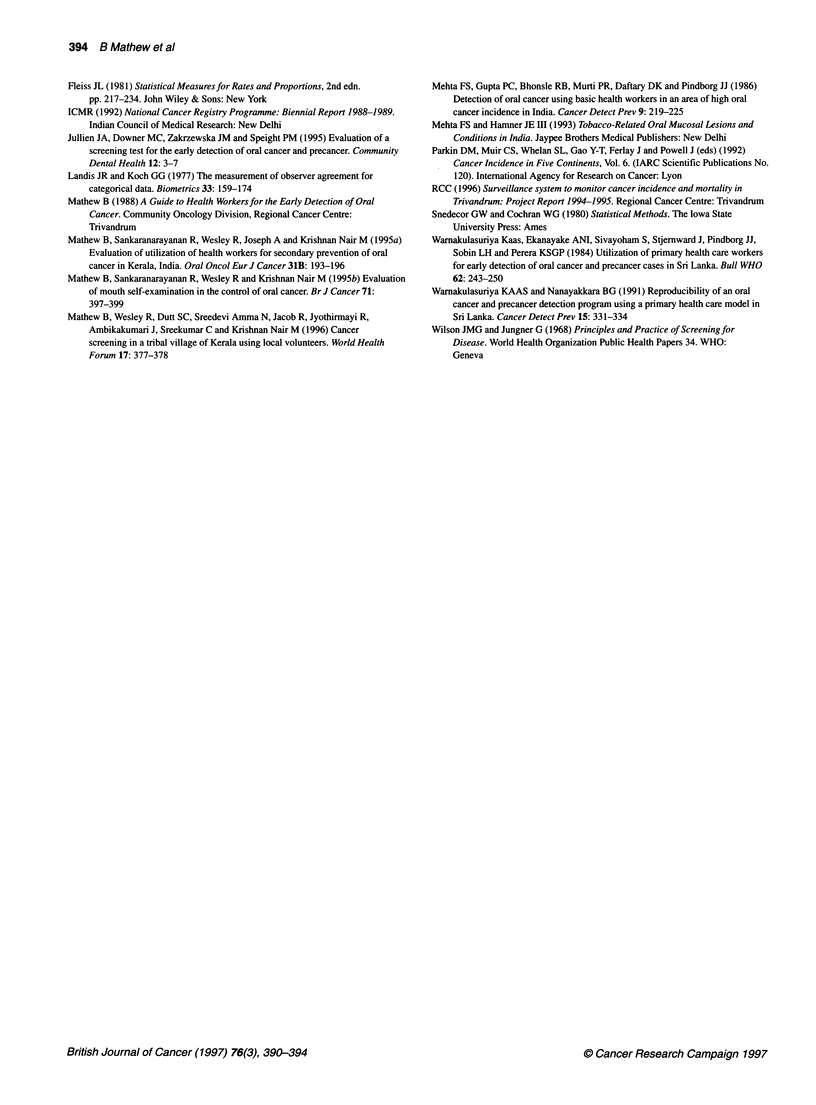# Reproducibility and validity of oral visual inspection by trained health workers in the detection of oral precancer and cancer.

**DOI:** 10.1038/bjc.1997.396

**Published:** 1997

**Authors:** B. Mathew, R. Sankaranarayanan, K. B. Sunilkumar, B. Kuruvila, P. Pisani, M. K. Nair

**Affiliations:** Regional Cancer Centre, Medical College Campus, Kerala, India.

## Abstract

A randomized intervention trial is in progress in Kerala, India, to evaluate the effectiveness of oral visual inspection by trained health workers (HWs) in the prevention of oral cancer. Fourteen health workers with college graduation as the basic qualification were trained in oral visual inspection to identify oral cancers and precancers among the participants of the screening trial and to refer them for further confirmation and management. The aim of the present study was to evaluate the reproducibility and validity of the screening test provided by the health worker against the reference oral visual findings of three physicians. A total of 2069 subjects who had already been examined were re-examined by the health workers and physicians. The sensitivity and the specificity of the oral visual inspection were 94.3% and 99.3% respectively. There was moderate agreement between the findings of the initial and the repeat mouth examinations carried out by the health workers, which were on average 6 months apart. There was almost perfect agreement (kappa = 0.85) between the findings of the health workers and the physicians in identifying the different types of oral precancerous lesions. The findings of our study indicate that it is possible to train resource persons to perform the oral cancer screening test as accurately as doctors, although experience appears to be a crucial component of health workers' accuracy. The efficacy of such an approach to reduce the incidence of and mortality from oral cancer, however, remains to be proven.


British Journal of Cancer (1997) 76(3), 390–394
© 1997 Cancer Research Campaign

### Reproducibility and validity of oral visual inspection by trained health workers in the detection of oral precancer and cancer

**B Mathew[1], R Sankaranarayanan[2], KB Sunilkumar[3], B Kuruvila[3], P Pisani[2] and M Krishnan Nair[1]**

[1]Regional Cancer Centre, Medical College Campus, Trivandrum 695011, Kerala, India; [2]Unit of Descriptive Epidemiology, International Agency for Research on Cancer, 150 cours Albert Thomas, 69372 Lyon cedex 08, France; [3]Trivandrum Oral Cancer Screening Project, Mangalapuram, Trivandrum 695 313, Kerala, India

**Summary** A randomized intervention trial is in progress in Kerala, India, to evaluate the effectiveness of oral visual inspection by trained health workers (HWs) in the prevention of oral cancer. Fourteen health workers with college graduation as the basic qualification were trained in oral visual inspection to identify oral cancers and precancers among the participants of the screening trial and to refer them for further confirmation and management. The aim of the present study was to evaluate the reproducibility and validity of the screening test provided by the health worker against the reference oral visual findings of three physicians. A total of 2069 subjects who had already been examined were re-examined by the health workers and physicians. The sensitivity and the specificity of the oral visual inspection were 94.3% and 99.3% respectively. There was moderate agreement between the findings of the initial and the repeat mouth examinations carried out by the health workers, which were on average 6 months apart. There was almost perfect agreement (kappa = 0.85) between the findings of the health workers and the physicians in identifying the different types of oral precancerous lesions. The findings of our study indicate that it is possible to train resource persons to perform the oral cancer screening test as accurately as doctors, although experience appears to be a crucial component of health workers' accuracy. The efficacy of such an approach to reduce the incidence of and mortality from oral cancer, however, remains to be proven.

**Keywords:** oral cancer; screening; validity; reproducibility; agreement; trained workers

Oral cancer is the most common malignant neoplasm among men and the third most common among women in several regions of India (Parkin et al, 1992; ICMR, 1992). It accounts for 10–25% of all male [average annual world age-adjusted incidence rate (AAR) 7–24 out of 100 000 men] and 8–15% of female cancers (AAR 3–13 out of 100 000 women). The highest worldwide incidence rate for oral cancer among women occurs in Bangalore, India.

Although oral cancer is amenable to primary and secondary prevention, this potential has not yet fully materialized, even in countries where it is the most common cancer. Preventive service delivery models, using the primary health care workers of the government health services, have been shown to be feasible in experimental settings in India (Mehta et al, 1986; Anantha et al, 1995) and in Sri Lanka (Warnakulasuriya et al, 1984; Warnakulasuriya and Nanayakkara, 1991); although this did not result in more staff being made available in practice (Mathew et al, 1995*a*). The feasibility of using voluntary health workers in the detection of oral cancer has also been demonstrated (Mathew et al, 1995*b*, 1996). To date, no randomized intervention study has been performed to evaluate the efficacy of primary and secondary prevention by health workers in reducing incidence and mortality from oral cancer.

*Received 29 October, 1996*
*Revised 23 January 1997*
*Accepted 24 February 1997*

*Correspondence to:* R Sankaranarayanan

Currently, we are evaluating a model of oral cancer screening in which the screening test, oral visual inspection, is administered by trained health workers (HWs) in a community-based randomized intervention trial in Trivandrum district, Kerala, India. Oral cancer accounts for one-fifth of all male (AAR 19.0 out of 100 000 men during 1991–93) and one-tenth of female cancers (AAR 8.5 out of 100 000 women) in this region (RCC, 1996). This study, initiated in October 1995, aims to recruit 90 000 persons aged 35–64 years resident in 13 panchayaths, which are the randomization units. A panchayath is a rural administrative structure with a total population of 30 000–40 000 people. The eligible subjects in seven intervention panchayaths are offered oral visual inspection by the HWs and those resident in the remaining six panchayaths serve as the control group.

A total of three screening examinations at 3-yearly intervals are planned for participants in the intervention panchayaths. Those in the control panchayaths are only enumerated for their sociodemographic and habit details, and no planned intervention is provided for them; 32 000 subjects have been recruited into the study since September 1996. Here, we discuss the validity and repeatability of the oral visual inspection provided by the HWs of this study.

### MATERIALS AND METHODS

Six (three male and three female) health workers, with college graduation as the basic qualification and resident in the study area, were identified for each study panchayath. Thus, 42 health workers from the intervention and 36 from the control panchayaths were selected for training. They were trained (1) to systematically enumerate households and individuals using a household form, (2) to interview the eligible subjects to elicit and record information on sociodemographic factors, tobacco and alcohol habits, past and present medical history and (3) to record height, weight and blood pressure.

The HWs for the intervention panchayaths ($n$ = 42) were additionally trained in the epidemiology, clinical features, diagnosis, investigation and management of oral precancers and cancers to enable them to carry out the following: (1) to perform systematic visual inspection of the buccal and labial mucosa, gingivae, bucco alveolar sulci, tongue, palate and floor of mouth, under adequate light and using two disposable wooden spatulas; (2) to identify homogeneous leucoplakia, ulcerated leucoplakia, verrucous leucoplakia, nodular leucoplakia, erythroplakia, submucous fibrosis and ulcers and growths suggestive of oral cancer and to refer them for further examination and management; (3) to perform physical examination of the neck in cases with oral cancer; and (4) to advise subjects with tobacco and alcohol habits to stop these practices. HWs for enumeration of subjects in control panchayaths were not trained on oral visual inspection and detection of lesions.

The training sessions, spread over a period of 6 weeks in November–December 1995, composed of lectures, practical demonstrations and field work, were conducted by a faculty consisting of a dentist, an epidemiologist and clinical oncologists from the Regional Cancer Centre (RCC), Trivandrum, and demographers and sociologists from the census department, Government of Kerala. A published guide entitled '*Tobacco-Related Oral Mucosal Lesions and Conditions in India*' (Mehta and Hamner, 1993) and a training manual prepared by the Community Oncology Division of the RCC in the vernacular Malayalam language for health workers in Kerala (Mathew, 1988) were used as the resource manuals for training. These provide descriptive and photographic documentation to identify the different types of oral lesions.

At the end of training sessions, written and practical tests were conducted to identify the best performing HWs. Based on the results in the test, two HWs (one male and one female) were finally selected for each panchayath. Thus, we had 14 HWs for the intervention panchayaths and 12 HWs for control panchayaths. The rest of the trained HWs have been kept in a reserve list for employment in case a HW leaves the project.

**Table 1** Comparison of the outcome of oral visual inspection by health workers at the initial and repeat screening examinations

| | Health worker findings (2) | | |
|---|---|---|---|
| **Health worker findings (1)** | **Positive** | **Negative** | **Total** |
| Positive | 182 | 198 | 380 |
| Negative | 47 | 1638 | 1685 |
| Total | 229 | 1836 | 2065 |

Kappa = 0.53, $z$ = 74.25.

Routine enumeration and examination of the subjects by house visits started soon after the training, in late December 1995. By May 1996, 9000 individuals in the intervention panchayaths had been examined by the HWs. One male and one female HW visited households in each intervention panchayath; the female HW was responsible for interviewing and for providing the screening test for female participants, and the male HW was responsible for the male participants of the study, for cultural reasons.

Identification of one or more of the following lesions resulted in a positive screening test: homogeneous leucoplakia, ulcerated leucoplakia, verrucous leucoplakia, erythroplakia, nodular leucoplakia, submucous fibrosis and oral cancer. Subjects identified with any of the above lesions were referred for examination by clinicians in regularly scheduled field clinics. Apparently normal mucosa and other non-referable conditions (e.g. lichen planus, aphthous ulcers, geographical tongue, etc.) resulted in a negative screening test.

The validity and repeatability of the oral visual inspection by the HWs was studied in the 2nd and 3rd weeks of May 1996 by re-examining 2069 (678 men and 1391 women) eligible subjects from among the 9000 subjects already recruited by the HWs during January–May 1996. These subjects were selected by choosing densely inhabited areas to allow re-examination of as many subjects as possible in 2 weeks. The time lag between the initial and the repeat examination by the HWs ranged from 1 to 6 months. The repeat examination on each subject was carried out by the HW who had performed the initial examination. All 14 HWs involved in providing oral cancer screening participated in the initial and repeat examinations.

**Table 2** Distribution and agreement by type of lesions on oral visual inspection by health workers and doctors

| | Doctors' findings | | | | | | | |
|---|---|---|---|---|---|---|---|---|
| **HW finding** | **Normal** | **HP** | **UP** | **VP** | **EP** | **SMF** | **Cancer** | **Total** |
| Normal | 1826 | 7 | 1 | 1 | 0 | 3 | 0 | 1838 |
| HP | 22 | 108 | 5 | 0 | 5 | 0 | 0 | 140 |
| UP | 0 | 2 | 10 | 0 | 1 | 0 | 0 | 13 |
| VP | 0 | 0 | 0 | 1 | 0 | 0 | 0 | 1 |
| EP | 6 | 3 | 4 | 0 | 30 | 0 | 0 | 43 |
| SMF | 1 | 0 | 0 | 0 | 0 | 30 | 0 | 31 |
| Cancer | 2 | 0 | 0 | 0 | 0 | 0 | 1 | 3 |
| Total | 1857 | 120 | 20 | 2 | 36 | 33 | 1 | 2069 |

HP, homogeneous leucoplakia; UP, ulcerated leucoplakia; VP, verrucous leucoplakia; EP, erythroplakia; SMF, submucous fibrosis. Kappa = 0.85, $z$ = 53.22.

© Cancer Research Campaign 1997

All subjects were aged 35–64 years, the mean age being 47.7 (s.d. 9.1) years. The HWs and doctors visited them in their homes, and the HWs performed the repeat screening test and recorded the findings of oral visual inspection. This was immediately followed by an independent examination of the same subject by the one of the three physicians (BM, KBS, RS) who had provided the reference test ('gold standard'): one of the physicians (BM) provided the reference test for 699 subjects, the other two provided the reference test for 681 (KBS) and 689 (RS) subjects

The reproducibility of HWs' examinations and their agreement with medical officers' outcome was measured using kappa statistics (Fleiss, 1981). Kappa values of 0.4–0.6 represent moderate agreement, 0.6–0.8 substantial agreement and 0.8–1.0 almost perfect agreement (Landis and Koch, 1977). The sensitivity and specificity of the oral visual inspections by the HWs were calculated by pooling all the results as well as by stratification according to HW male–female team, sex and age. The difference between proportions and confidence intervals was tested and computed by means of the normal approximation applied to the arc sin transformed values (Snedecor and Cochran, 1980).

A correlation between visual inspection and pathological findings is not possible as biopsy has not been performed for most cases. Biopsy is performed for cases of nodular leucoplakias, erythroplakias and suspicious growths only, and this is currently being undertaken.

### RESULTS

The concordance between the results of oral examination by HW at the time of initial recruitment of subjects (December 1995–May 1996) and at re-examination in mid May 1996 in terms of referable and non-referable lesions is shown in Table 1. The agreement between the two findings was moderate as evidenced by the kappa value of 0.53. Fewer cases were designated positive on the second testing ($n$ = 229) than on the first ($n$ = 380), perhaps as a result of accumulation of experience.

Findings of oral visual inspection by the HWs and the doctors are compared in Tables 2 and 3. When looking at single types of lesions, agreement was also very good, as evidenced by the 'kappa' statistics: for homogeneous leucoplakia, kappa = 0.84 ($P < 0.001$); erythroplakia, kappa = 0.81 ($P < 0.001$); ulcerated leucoplakia; kappa = 0.83 ($P < 0.001$); and submucous fibrosis, kappa = 0.94 ($P < 0.001$). The maximum agreement was found for diagnosis of submucous fibrosis. The overall agreement between the HWs and the doctors in identifying the different types of lesions was very good (kappa = 0.85, $P < 0.001$).

There were 212 (10.3%) subjects with true positive lesions (as classified by the three physicians) among the 2069 re-examined; 12 subjects with true positive lesions were missed by the HWs (Table 3). There was almost perfect agreement between the physicians and the HWs in designating subjects positive or negative (kappa = 0.89, $P < 0.001$). The overall sensitivity was 94.3%, specificity 99.3% and positive predictive value 86.6%. The values of sensitivity did not differ significantly by age (96.8% for those aged less than 50 years and 92.3% for those 50 years and above) and sex (95.4% in men and 93.2% in women). However, the specificities were significantly different when examined by sex (96.7% in men and 93.2% in women) and age (92.3% in those aged < 50 years and 97.3 in those aged 50+ years) ($P < 0.01$); however, this seems to be a reflection of large numbers of negative findings, and hence, for all practical purposes, the specificities were comparable.

There was no significant difference in sensitivity, specificity and predictive values of the screening test provided by the seven HW teams; sensitivity varied from 88.2% to 97.5% and specificity from 96.9% to 98.9%.

Comparability of findings of oral visual inspection by the three physicians who provided the reference test was examined by studying the agreement in the results of mouth examination of 100 subjects. There was almost perfect agreement among the findings of the three physicians, as indicated by the overall kappa value of 0.85 ($P < 0.001$).

**Table 3** Validity of health workers findings against doctors findings, both overall and by sex and age group of the participants

| HW findings | Doctor's findings | | Sensitivity (95% CI) | Specificity (95% CI) | PPV | NPV |
|---|---|---|---|---|---|---|
| | Positive | Negative | | | | |
| Overall | | | | | | |
| Positive | 200 | 31 | 94.3 | 98.3 | 86.6 | 99.3 |
| Negative | 12 | 1826 | (90.4–96.7) | (97.6–98.8) | | |
| Men | | | | | | |
| Positive | 104 | 19 | 95.4 | 96.7 | 84.6 | 99.1 |
| Negative | 5 | 550 | (89.7–98.0) | (94.8–97.9) | | |
| Women | | | | | | |
| Positive | 96 | 12 | 93.2 | 99.1 | 88.9 | 99.5 |
| Negative | 7 | 1276 | (86.6–96.7) | (98.4–99.5) | | |
| Age < 50 years | | | | | | |
| Positive | 92 | 11 | 96.8 | 92.3 | 89.3 | 99.7 |
| Negative | 3 | 1112 | (91.1–98.9) | (98.3–99.5) | | |
| Age > 50 years | | | | | | |
| Positive | 108 | 20 | 92.3 | 97.3 | 84.4 | 98.8 |
| Negative | 9 | 714 | (86.0–95.9) | (95.8–98.2) | | |

PPV, positive predictive value; NPV, negative predictive value.

© Cancer Research Campaign 1997

### DISCUSSION

Although oral cancer and oral visual inspection meet most of the criteria of Wilson and Jungner (1968) for a suitable disease and a suitable screening test, there is no evidence that screening for oral cancer is effective in reducing incidence and mortality from disease. So far, this issue has not been addressed in randomized studies. To date, only one study has investigated the role of screening in oral cancer control (Fernandez Garrote et al, 1995). As oral cancer is an important problem in India and South and South-East Asia and in certain other regions of the world, and as the mortality from oral cancer has either remained stable or increased in different regions (Coleman et al, 1993), there is a need to evaluate oral visual inspection for its efficacy in controlling oral cancer.

The objective of the present study was to assess the ability of HWs employed in the intervention panchayaths of our trial to identify referable lesions, as satisfactory performance of the screening test is an important aspect of the screening programme. Hence, we wished to validate the performance of the screening test provided by HWs in terms of sensitivity and specificity and their ability to identify the different types of intraoral lesions. If we had found the test characteristics to be unacceptable (sensitivity <90%, specificity <95%), we had plans to retrain the HWs by organizing another intensive session. However, the high values of sensitivity as well as specificity, by both pooled and stratified analysis, and the high level of agreement between them and the physicians in identifying the different lesions reveal that our HWs have acquired good skills in providing oral visual inspection.

Health workers (HWs) employed by the government health services have been used in previous studies to address the feasibility of using health care auxiliary personnel in the control of oral cancer in India and Sri Lanka (Warnakulasuriya et al, 1984; Mehta et al, 1986; Warnakulasuriya and Nanayakkara, 1991). These studies have proved that trained HWs were capable of carrying out a proper mouth examination and of identifying lesions.

In the earlier Sri Lankan study, 29 215 (33.6%) of the 87 277 eligible population aged 20 years and above were examined by HWs (Warnakulasuriya et al, 1984). Some 565 of the 1220 referred subjects were further examined by dentists: 338 (60%) had oral precancers, 14 (2%) had cancers and 213 (38%) had benign or no lesions. Almost similar results were obtained when this model was reproduced in another region of Sri Lanka (Warnakulasuriya and Nanayakkara, 1991).

The performance of mouth examination by HWs has been assessed in a previous study in Ernakulam district, Kerala, India, by Mehta et al (1986). Re-examination of a sample of 1921 subjects by dentists, from a total of 39 331 individuals examined by HWs, revealed that visual inspection by HWs had a sensitivity of 56%, specificity of 98% and a positive predictive value of 30% to detect one or more of the following lesions: nodular leucoplakia, erythroplakia, submucous fibrosis and growths/ulcers suggestive of oral cancer. Our results compare favourably to those reported by Mehta and co-workers (1986).

A long-term feasibility study to evaluate the role of government HWs in the early detection of oral cancer in Trivandrum district failed to motivate the HWs to provide oral visual inspection and to refer subjects appropriately (Mathew et al, 1995*a*). More than 90% of the trained HWs did not participate in the programme at all.

Two recent studies from the UK reported sensitivity of 71–74% and specificity of 99% for oral visual inspection administered by dentists (Downer et al, 1995; Jullien et al, 1995). Dentists in the Cuban health services provide the screening test in their national oral cancer screening programme (Fernandez Garrote et al, 1995). During the period 1984–90, 12 990 677 examinations were performed and 30 244 (0.23%) subjects were referred; 8703 (28.8%) complied with referral. Among them, 8.1% had oral cancer, 37% oral precancer and 54.9% had either benign or no lesions. Thus, the false-positive referrals in reported studies of oral cancer screening varied from 20% to 55%.

In our study, the agreement between the results of oral examination by HWs at the time of accrual of subjects and at repeat examination was only moderate for several reasons. Repeat examinations on each participant were performed by the same HW; therefore, variation between HWs is not a cause for disagreement between the initial and repeat examinations.

The two examinations by the HWs were performed on average 6 months apart, during which time HWs had accumulated further experience in identifying lesions; it would not be surprising if their performance improved. Some subjects had either stopped or reduced tobacco and/or alcohol habits in response to the intervention programme, and few subjects were consuming vitamin A; hence, their lesions may have either disappeared or transformed into another type. Lesions in some subjects might have also progressed. Hence, some variation in the findings on the two occasions is to be expected. Conversely, the HWs second examination and that of the doctors were all performed at the same time; they were examining the same subjects and the same lesions.

We believe that a critical appraisal of physical examination of the mouth for its efficacy in reducing mortality from oral cancer is important in the consideration of its inclusion as part of the primary health care provided by the primary HWs (who are already overloaded with several responsibilities) in regions where oral cancer is common. The present investigation confirms the observation that HWs can be adequately trained to identify oral lesions. The ultimate aim of our intervention trial is to evaluate a primary health care approach with suitably trained HWs to determine whether screening of this nature reduces mortality from oral cancer.

### ACKNOWLEDGEMENTS

The Trivandrum Oral Cancer Screening Trial is supported by a generous grant from the Association of International Cancer Research (AICR), St Andrews, Scotland, UK; their valuable continuing support is gratefully acknowledged. The authors thank Dr DM Parkin, Chief, Unit of Descriptive Epidemiology, International Agency for Research on Cancer, for his critical comments on a draft version of this paper and Mrs E. Bayle for her help in preparing this manuscript.

### REFERENCES

Anantha N, Nandakumar N, Vishwanath N, Venkatesh T, Pallad YG, Manjunath P, Kumar DR, Murthy SGS, Shivashankaraiah and Dayananda CS (1995) Efficacy of an anti-tobacco community education program in India. *Cancer Causes Control* **6**: 119–129

Coleman MP, Esteve J, Damiecki P, Arslan A and Renard H (1993) *Trends in Cancer Incidence and Mortality*. IARC Scientific Publications No. 121. IARC: Lyon

Downer MC, Evans AW, Hughes Hallet CM, Jullien JA, Speight PM and Zakrzewska JM (1995) Evaluation of screening for oral cancer and precancer in a company headquarters. *Community Dent Oral Epidemiol* **23**: 84–88

Fernandez Garrote L, Sankaranarayanan R, Lence Anta JJ, Rodriguez Salva A and Parkin DM (1995) An evaluation of the oral cancer screening program in Cuba. *Epidemiology* **6**: 428–431

© Cancer Research Campaign 1997 

Fleiss JL (1981) *Statistical Measures for Rates and Proportions*, 2nd edn. pp. 217–234. John Wiley & Sons: New York

ICMR (1992) *National Cancer Registry Programme: Biennial Report 1988–1989*. Indian Council of Medical Research: New Delhi

Jullien JA, Downer MC, Zakrzewska JM and Speight PM (1995) Evaluation of a screening test for the early detection of oral cancer and precancer. *Community Dental Health* **12**: 3–7

Landis JR and Koch GG (1977) The measurement of observer agreement for categorical data. *Biometrics* **33**: 159–174

Mathew B (1988) *A Guide to Health Workers for the Early Detection of Oral Cancer*. Community Oncology Division, Regional Cancer Centre: Trivandrum

Mathew B, Sankaranarayanan R, Wesley R, Joseph A and Krishnan Nair M (1995*a*) Evaluation of utilization of health workers for secondary prevention of oral cancer in Kerala, India. *Oral Oncol Eur J Cancer* **31B**: 193–196

Mathew B, Sankaranarayanan R, Wesley R and Krishnan Nair M (1995*b*) Evaluation of mouth self-examination in the control of oral cancer. *Br J Cancer* **71**: 397–399

Mathew B, Wesley R, Dutt SC, Sreedevi Amma N, Jacob R, Jyothirmayi R, Ambikakumari J, Sreekumar C and Krishnan Nair M (1996) Cancer screening in a tribal village of Kerala using local volunteers. *World Health Forum* **17**: 377–378

Mehta FS, Gupta PC, Bhonsle RB, Murti PR, Daftary DK and Pindborg JJ (1986) Detection of oral cancer using basic health workers in an area of high oral cancer incidence in India. *Cancer Detect Prev* **9**: 219–225

Mehta FS and Hamner JE III (1993) *Tobacco-Related Oral Mucosal Lesions and Conditions in India*. Jaypee Brothers Medical Publishers: New Delhi

Parkin DM, Muir CS, Whelan SL, Gao Y-T, Ferlay J and Powell J (eds) (1992) *Cancer Incidence in Five Continents*, Vol. 6. (IARC Scientific Publications No. 120). International Agency for Research on Cancer: Lyon

RCC (1996) *Surveillance system to monitor cancer incidence and mortality in Trivandrum: Project Report 1994–1995*. Regional Cancer Centre: Trivandrum

Snedecor GW and Cochran WG (1980) *Statistical Methods*. The Iowa State University Press: Ames

Warnakulasuriya Kaas, Ekanayake ANI, Sivayoham S, Stjernward J, Pindborg JJ, Sobin LH and Perera KSGP (1984) Utilization of primary health care workers for early detection of oral cancer and precancer cases in Sri Lanka. *Bull WHO* **62**: 243–250

Warnakulasuriya KAAS and Nanayakkara BG (1991) Reproducibility of an oral cancer and precancer detection program using a primary health care model in Sri Lanka. *Cancer Detect Prev* **15**: 331–334

Wilson JMG and Jungner G (1968) *Principles and Practice of Screening for Disease*. World Health Organization Public Health Papers 34. WHO: Geneva

© Cancer Research Campaign 1997